# Oral health knowledge, perceptions and attitudes of pregnant women in Sub-Saharan Africa: a systematic review

**DOI:** 10.1186/s12903-025-06249-y

**Published:** 2025-06-06

**Authors:** Torojah Mayaline Williams, Adetola Emmanuel Babalola, Olubusayo Bolarinwa, Victor Adeyanju Somoye, Oluwaseun Akinola Azeez, Olayinka Julianah Onasanya, Victor Miracle Johnson, Adaeze Favour Egemonye

**Affiliations:** 1 Institute for Health and Equity, Medical College of Wisconsin, Milwaukee, WI USA; 2 Faculty of Dentistry, College of Medicine, University of Ibadan, Ibadan, Nigeria; 3 Johns Hopkins University, Maryland, USA; 4 College of Medicine, University of Lagos, Lagos, Nigeria

**Keywords:** Knowledge, Perception, Attitude, Oral health, Pregnancy, Women, Africa

## Abstract

**Background:**

This systematic review aimed to assess pregnant women’s knowledge, perceptions, and attitudes toward oral health changes in sub-Saharan Africa (SSA) and examine their oral health-seeking behavior during pregnancy.

**Methods:**

A comprehensive search was conducted across PubMed, Google Scholar, African Journals Online (AJOL), the Directory of Open Access Journals (DOAJ), and the Cochrane Library. Both Medical Subject Headings (MeSH) and free-text terms related to oral health, knowledge, attitudes, perceptions, antenatal care, pregnancy, and Africa were used. Boolean operators (“AND,” “NOT,” and “OR”) refined the search strategy. Two independent reviewers screened studies and extracted data using Rayyan software, with a third reviewer resolving conflicts. The Robins-E tool assessed the risk of bias.

**Results:**

Fifteen of seventy-five studies initially identified met the inclusion criteria after full-text screening. Most employed a cross-sectional design. Findings revealed low oral health-seeking behavior among pregnant women in Africa, attributed to factors such as negative dental experiences, concerns about harm to the unborn child, and the belief that dental care is unnecessary. Many women visited dental clinics only when they noticed significant oral health changes.

**Conclusions:**

The reviewed studies demonstrated a low risk of bias and consistent findings. Pregnancy is a critical period, and poor oral health can adversely affect birth outcomes. Insufficient knowledge and misconceptions deter women from seeking dental care during pregnancy. To address this, targeted oral health education must improve awareness and overcome barriers to seeking care.

**Supplementary Information:**

The online version contains supplementary material available at 10.1186/s12903-025-06249-y.

## Introduction

Pregnancy is a period marked by significant physiological changes that affect various systems in the body, including oral health [1]. The associated hormonal fluctuations, like increases in estrogen and progesterone levels, alter the body’s inflammatory response, making pregnant women very susceptible to oral conditions like gingivitis, periodontitis, and pyogenic granuloma [1]. Gingivitis affects 60–75% of pregnant women globally and is characterized by gingival swelling and bleeding [2]. If left untreated, gingivitis can progress to periodontitis, a severe infection leading to tooth loss. Pyogenic granulomas can also result from heightened inflammatory responses, typically occurring in the second or third trimester [3]. These conditions underscore the importance of maintaining proper oral hygiene throughout pregnancy.

Oral health management during pregnancy varies globally, with some regions integrating it effectively into antenatal care [4]. In high-income countries, pregnant women receive education on oral hygiene as part of routine prenatal care, recognizing the link between maternal oral health and pregnancy outcomes [5, 6]. Studies like the one carried out by Kaur et al. [7] have shown that poor oral health in pregnancy is associated with adverse outcomes like preterm birth and low birth weight, highlighting the importance of integrating oral health into prenatal care. Despite these connections, there are still gaps in consistent care delivery in many regions.

The state of maternal oral healthcare reflects broader healthcare disparities. For instance, Adam et al. [8] noted that countries like Nigeria and Sudan provide inadequate awareness and education about oral health during pregnancy. The disparity in access to oral healthcare between urban and rural regions further highlights these challenges, leaving many women vulnerable to preventable oral health conditions. Moreover, neglecting oral health during pregnancy can have significant consequences.

In many African settings, this neglect worsens existing health disparities, particularly among low-income populations [9]. As a result, it burdens healthcare professionals to take decisive steps to address these gaps. However, oral health education is often not prioritized in antenatal care services, leading to missed opportunities for prevention and treatment [8]. Furthermore, comprehensive research on these cultural and healthcare barriers is essential for developing targeted interventions and improving maternal and fetal health outcomes across Africa. Understanding these women’s knowledge gaps and misconceptions about oral health can help shape more effective healthcare strategies, improving maternal and neonatal outcomes.

This systematic review aims to provide a narrative synthesis of oral health knowledge, attitudes, and perceptions of pregnant women in sub-Saharan Africa (SSA) while providing recommendations to optimize dental care during and after ante-natal care visits.

## Methods

### Study protocol and registration

This systematic review was reported following the guidelines of the Preferred Reporting Items for Systematic Reviews and Meta-Analyses (PRISMA) [10]. This systematic review was registered with PROSPERO (International Prospective Register of Systematic Reviews) under the number CRD42024593615. The protocol stated the research questions, objectives, inclusion and exclusion criteria, and other preparatory details for this systematic review.

### Study eligibility criteria

We utilized a PEO (Population, Exposure, and Outcome) framework and constructed a detailed guide to the studies to be included. Our population included pregnant women in sub-Saharan Africa. Exposure includes the knowledge, perceptions, and attitudes toward oral health, and our outcomes assessed pregnant women’s oral health knowledge, perceptions, and behavioral practices. Studies could range from simple observational studies to complex interventional studies.

### Inclusion and exclusion criteria

We included studies conducted on individuals residing in SSA (sub-Saharan Africa). Additional filters were added for female participants. We focused on original research studies, including cross-sectional, cohort, case-control, and intervention studies, report on the knowledge, perceptions, attitudes, or behaviors of pregnant women regarding oral health changes. Studies were excluded if they lacked English language versions. Finally, articles with partial, summarised, or incomplete reports such as conference presentations, proceedings, and abstracts were excluded.

### Search strategy

To ensure relevant studies addressing knowledge, perception, and attitudes of pregnant women to oral health changes during pregnancy in Africa were included in this systematic review, we conducted a systematic search across PubMed, Google Scholar, African Journals Online (AJOL), Directory of Open Access Journals (DOAJ), and Cochrane Library. We used a combination of Medical Subject Headings (MeSH) and free-text terms related to Oral Health, knowledge, attitudes, behaviors, perceptions, ante-natal, pregnancy, and Africa. Boolean operators (“AND”, “NOT”, and “OR”) were used to refine the search strategy and ensure comprehensive coverage. The search covered studies published from the inception of each database to 30th March, 2025.

### Data extraction

All studies (CSV/bib.text/ris/Revman/Endnote) were imported on the Rayyan software [11], screened out for duplicates, and managed by two independent reviewers, T.M.W and A.E.B. The studies had their titles and abstracts screened, and any concerns were resolved by a third independent reviewer (O.B). A final full-text screening and data extraction were included (Authors and year, study design, sample size, location, and main findings).

### Risk of bias assessment

**Fig. 1:**
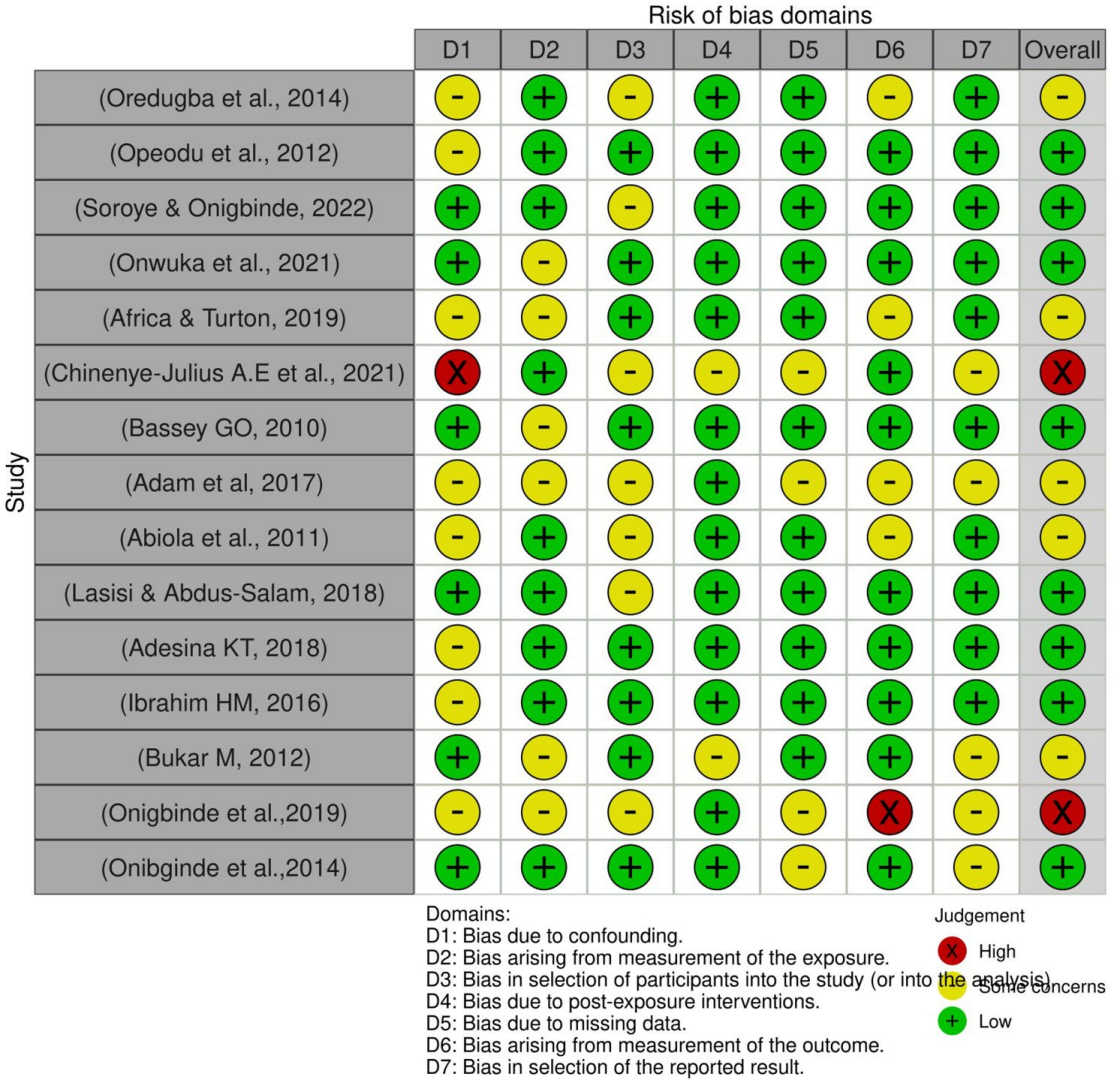
Risk of bias assessment

T. W. and A.B. used the ROBIN-E (for observational studies) tool to assess the Quality of the included studies (Fig. 1). The ROBIN-E tool analyzes the risk of bias across seven domains, including confounding, measurement of exposure, selection of participants, post-exposure interventions, missing data, measurement of outcome, and selection of reported results [12]. The final classification depends on the highest risk rating across the domains. In our analysis, low risk was given when all domains are rated as low risk or, at most, one domain is moderate risk. Moderate risk is when one or more domains are rated as moderate risk, but no domain is serious or critical. High risk was given when at least one domain is rated as high risk Most of the studies had a low risk of bias, and some had a medium risk and a high risk of bias.

## Results

**Fig. 2:**
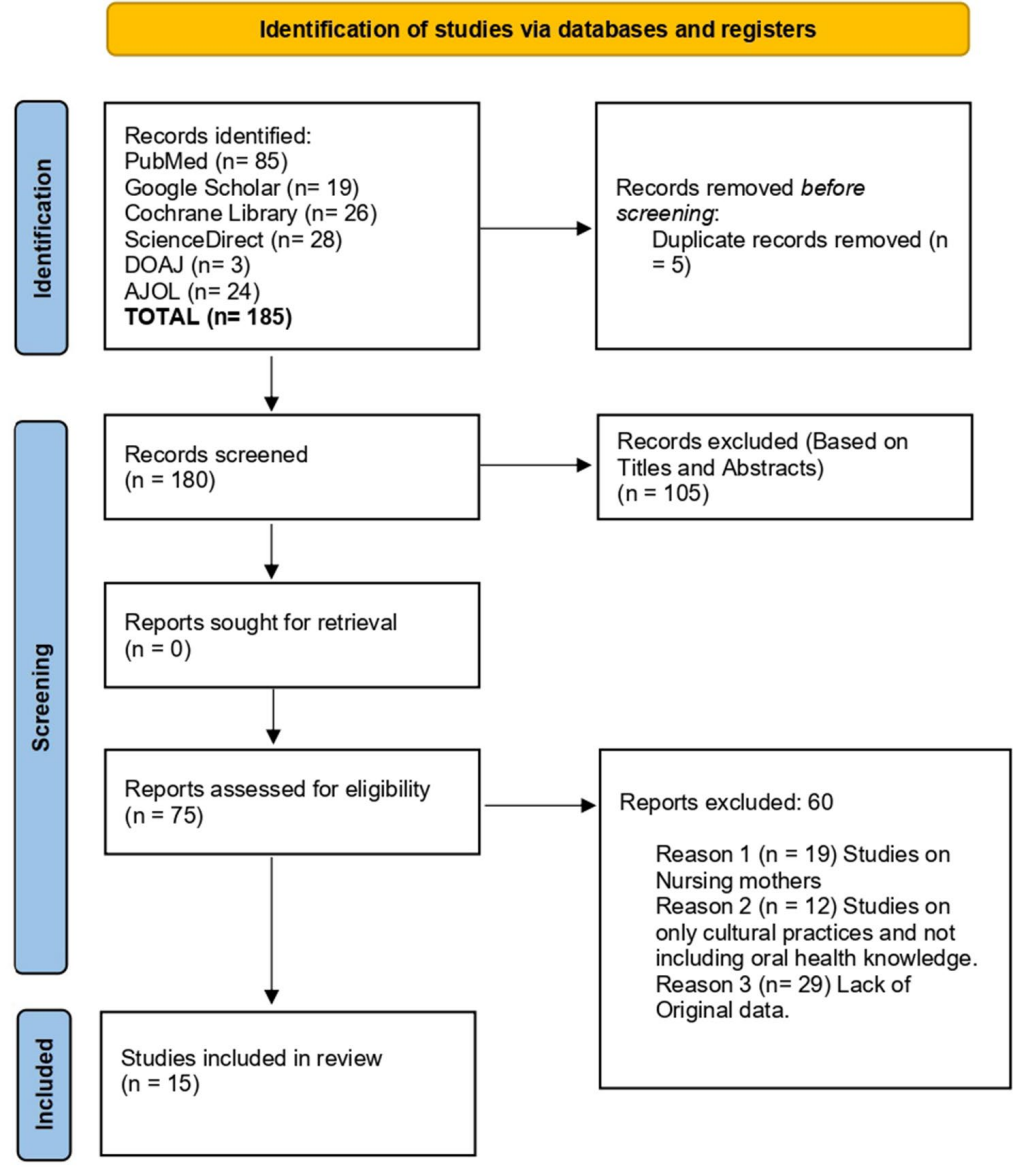
Prisma flow chart of included studies

The reviewed studies investigated African pregnant women’s knowledge, perceptions, and behaviors regarding oral health changes. Our initial search yielded 185 studies, five duplicates were removed, and an initial screening of abstracts and titles was done. Finally, 75 underwent full-text screening (Fig. 2). Fifteen studies were eligible for inclusion. All the study designs were cross-sectional. The total sample size for this systematic review was 4,639 individuals across Nigeria, Sudan, and South Africa. Study-specific characteristics and references are provided in Table 1.

**Table 1:** Table of individual included study characteristics: oral health knowledge, perceptions and attitudes of pregnant women in Sub-Saharan Africa: a systematic review

Author and year	Study design	Sample size	Location	Results
(Oredugba et al., 2014)	Cross-sectional study	115	Nigeria	The knowledge of oral health of the mothers who participated in this study is inadequate and therefore they will benefit from regular oral health education by all health professionals
(Opeodu et al., 2012)	Cohort study	384	Nigeria	There is a tendency for a reduction in gingival bleeding post-pregnancy and there is an urgent need for dental campaign geared towards the sensitization of women of child-bearing age as to the need to maintain optimal oral health
(Onigbinde et al., 2014)	Cohort study	415	Nigeria	Gestational age of pregnancy and dental visits have a definite impact on the periodontal status. Oral health education should be included as an integral part of antenatal care to increase women’s awareness.
(Onigbinde et al., 2019)	Cross-sectional study	426	Nigeria	Most respondents knew the importance of good oral hygiene but majority have negative attitude towards seeking dental care during pregnancy.
(Soroye & Onigbinde, 2022)	Self-administered questionnaires	151	Nigeria	Knowledge of pregnancy gingivitis is poor among participants
(Onwuka et al., 2021)	Descriptive cross-sectional study	350	Nigeria	There were knowledge gaps in the oral health of pregnant women. It is therefore important to provide oral health education for pregnant women during antenatal period in order to improve the health of the mother and her baby.
(Africa & Turton, 2019)	Cross-sectional study	443	KwaZulu-Natal, South Africa	Maintaining good oral hygiene before and during pregnancy is crucial for ensuring oral health
(Chinenye-Julius A.E et al., 2021)	Descriptive cross-sectional study	385	Ogun, Nigeria	Majority (56%) of the respondents had positive attitudes towards oral hygiene during pregnancy while 44% had negative attitude. Oral health education and workshops should be put in place to increase knowledge on advantages of maternal and child oral health
(Bassey GO et al., 2010.)	Questionnaire-based study	252	Calabar, Nigeria	It is recommended that oral health education be incorporated into the antenatal counseling of pregnant women in Nigeria.
(Adam et al., 2017)	Descriptive cross-sectional study	274	Nigeria	Majority of the respondents had poor knowledge of oral hygiene, despite the high awareness. There is a need for oral hygiene education of pregnant women during antenatal clinics
(Abiola et al., 2011)	Self-administered questionnaire-based survey	460	Nigeria	Important gaps in their oral health knowledge and practices. Hence, oral health education during antenatal care to educate women on the importance of maintaining good oral health is essential.
(Lasisi & Abdus-Salam, 2018)	Cohort study	81	Nigeria	Oral health disease prevention and health promotion is necessary to reduce the burden of gingival and periodontal disease burden
(Adesina KT et al., 2018)	Case control study	225 cases, 166 controls	Nigeria	The prevalence of oral complaints among the pregnant women was 19.1%. Gingivitis was more common among the pregnant women than the non-pregnant women, and more demonstrable on examination. Oral healthcare should be a component of the antenatal care in our environment
(Ibrahim HM et al., 2017)	An observational, cross-sectional, hospital-based study	384	Sudan	Findings suggest the need for oral health programmes as part of prenatal care for pregnant Sudanese women
(Bukar M et al., 2012)	Cross sectional study	294	Nigeria	Oral healthcare among the respondents was encouraging but the involvement of the dental surgeon in preventive oral care in pregnancy is rather dismal.

### Negative attitude toward seeking dental care during pregnancy

Four studies reported the harmful oral health practices and dental-seeking behaviors of pregnant women [13–16]. Onigbinde et al., in a cross-sectional study of 426 pregnant women, reported that 79% of the participants had a low oral health knowledge score, which translated to their behavioral practices [15]. Only 41.1% brushed twice daily, and their dental-seeking practices were also low, as only 19.2% visited the dentist during their pregnancy, although 50.7% had never visited the dentist [15]. Abiola et al. surveyed 460 pregnant women receiving antenatal care at the Lagos State University Teaching Hospital (LASUTH) [14]. It was reported that 23.4% of participants felt seeking dental care was unnecessary during pregnancy. It was also stated that 9.1% (42/453) of respondents deemed visits to the dentist as always unpleasant [14]. This sentiment may be from prior individual or second-hand negative experiences, i.e., from friends and relatives. The findings also reported that for every unit increase in educational class, poor oral health knowledge decreases by 83% [14].

Chinenye-Julius et al. reported in 2021 that 44% of respondents had negative oral health attitudes, and a third of the participants agreed that they did not need to visit the dentist because it was harmful to the unborn child [16]. Further analysis also found no significant association between trimester and oral health practices [15]. A critical practice that reflected the low dental-seeking behavior was the use of dental floss. A self-administered cross-sectional study designed in 2022 by Soroye and Onigbinde found that a low 19.4% used dental floss [13]. Three years earlier, in the same environment (Lagos, Nigeria) Onigbinde et al. had reported that only 12.4% used dental floss [15].

Most of the studies reported low dental-seeking behavior, consistent with findings from Oredugba et al., which found that only 7.97% of 109 antenatal care attending mothers had ever visited the dentist [17]. These findings are significantly associated with occupational status and education [17].

### Positive oral health-seeking behavior during pregnancy and treatment needs

The willingness of pregnant women to seek oral health care during pregnancy was reported in Sudan, Nigeria (Benin, Ogun state, and Calabar). Adam et al. reported that 75.9% of their descriptive cross-sectional study participants in Benin City were willing to visit the dentist immediately on account of dental caries, and 79.6% were willing to do the same on account of toothache [8]. A hospital-based observational, cross-sectional study conducted in Sudan also showed that only 20.0% of the 384 participants planned to visit a dentist [18].

A descriptive cross-sectional study conducted in primary health care centers in Ijebu North-east local government area in Ogun state by Chinenye-Juliuset al. revealed that most participants (65.5%) were willing to seek dental care during pregnancy because of any changes that may occur [16]. 83.3% of 252 respondents in the questionnaire-based study conducted in Calabar by Bassey. et al. were willing to have a dental consultation during pregnancy [19].

Onigbinde et al. in a cross-sectional study of 426 pregnant women in Lagos state, also reported on the willingness of pregnant women to undergo dental treatment after delivery, and 65.5% of respondents were willing to do so on account of periodontal disease [15]. Africa and Turton reported that of the 443 South African participants, 38 (8.5%), 65 (14.7%), and 26 (5.9%) of women had pregnancy epulis, oral lesions, and tooth mobility, respectively [20]. Comparatively, in a case-control study by Adesina et al., the study reported that the gingivitis was the most common oral condition, and the prevalence of dental complaints amongst 255 pregnant women living in north-central Nigeria was 19.1% [21].

### Self-reported oral health knowledge and oral hygiene habits

A study by Opeodu et al., involving 284 women in their third trimester, showed that 261 participants experienced bleeding during pregnancy, which decreased to 192 and 127 at the 6th and 14th weeks postpartum, respectively [22]. These findings suggest that pregnant women were already aware of pregnancy-associated dental conditions [22].

In a cohort study conducted among 81 pregnant women at Adeoyo Hospital in Ibadan, Nigeria, only 6.5% reported dental complaints. Of the participants, 40.7% had good oral hygiene scores, and 26% reported brushing twice daily [23]. Additionally, 6.5% had healthy periodontal status, while 10.4% had healthy gingival status [23]. Ibrahim et al. in a cross-sectional study involving 384 women in Sudan, reported that 88.1% of participants recognized toothbrushing as effective in preventing tooth decay, gum disease, and bad breath. In contrast, only 2.6% regarded it as simply a habit [18]. Most respondents (85.5%) reported brushing their teeth more than once a day, with 14.5% brushing once daily [18]. However, only 9.5% utilized other oral hygiene practices such as dental floss, toothpicks, miswak, or mouthwash [18].

In a cross-sectional study by Adam et al. in Benin, Nigeria, almost all the participants (99.3%) used toothbrushes, though only 2.2% used alternatives like baking soda or table salt [8]. Additionally, 73.4% replaced their toothbrushes every 1 to 3 months [8]. Most (99.3%) brushed with toothpaste, and 74.1% specifically used fluoride. While 60.7% cited removing germs and bacteria as their main reason for brushing, 24.3% mentioned preventing halitosis [8]. Soroye et al. reported that only a third of the respondents (33.8%) brushed twice daily [13]. The study also found that 1.3% (2 persons) thought it was normal to bleed from the gums while brushing [13].

In a study by Onwuka et al., 8.7% of the respondents had dental consultations in pregnancy for complaints such as toothache and decay (66.7%) and pain as well as swelling of the gum (33.3%) [24]. Bukar et al. found that women who were employed and had a low parity reported better oral care [25]. Contrarily, this differs from the findings of Onigbinde in 2014, who found no significant association between periodontal status and parity [26].

## Discussion

Pregnancy is when a woman experiences many changes in her body, from physiological to hormonal to dietary to emotional. Many epidemiological studies have established a link between oral health and adverse pregnancy outcomes [27, 28]. This systematic review looks at how women on the African continent have navigated changes in their oral health.

The studies reported negative or low oral health-seeking behavior among pregnant women, with reasons ranging from a dental visit being unpleasant, oral health being unnecessary to visit the dentist during pregnancy, to beliefs that it would harm their unborn child. Onigbinde et al., in a cross-sectional study with 426 participants, found that many pregnant women had low dental health knowledge, which translated to the oral health behavior they exhibited [15]. The reasons for not seeking dental services were similar to those of Naseem, M et al. [29], including lack of knowledge and value, negative oral health experiences, negative attitudes toward oral health professionals, and negative attitudes of dental staff toward pregnant women.

Studies examining pregnant women’s willingness to seek dental care during pregnancy revealed that most women will seek dental care only if there is a change from the norm, with symptoms such as a toothache or dental caries. Onwuka *et al.’s* study showed toothache as the most common dental complaint [24]. This further buttresses the point that pregnant women would only deem a visit to the dentist necessary if there is a change from what is considered normal.

Contrary to the findings of Onigbinde et al., which reported low dental health knowledge among pregnant women in Lagos, Nigeria [15], Opeodu et al., in Ibadan, Nigeria, showed results that suggested that the women were aware of oral health changes associated with pregnancy. The studies by Adams et al. in Benin, Nigeria [8] and Ibrahim et al. in Sudan [18] all found that the women engaged in toothbrushing as a way of maintaining oral hygiene and preventing tooth decay, gum disease, and bad breath, indicating that the women had an awareness of dental health. Similar studies [30, 31] have reported that most women believed that oral problems such as tooth decay and bleeding gums are part of the pregnancy changes, and this belief serves as a barrier to dental consultation. In a study by Hussein et al. in Somalia, 82.1% of participants were found to have periodontitis [32]. This study identified factors contributing to their oral health habits, such as low socioeconomic class, education, tobacco, chewing Miraa (*Catha Edulis* plant), and alcohol consumption [32].

All the studies in this systematic review recommended that oral health education be employed to gear more pregnant women toward dental health-seeking behavior. Onwuka et al. found that after counseling participants in their study, 60.3% agreed to have dental consultations during subsequent pregnancies [24]. Oral health education and workshops should be implemented to increase knowledge of the advantages of maternal and child oral health [4]. Incorporating oral health education into the information sessions at the antenatal clinics could go a long way in increasing oral health knowledge in pregnant women and correcting any misinformation or cultural beliefs associated with oral health changes and practices among pregnant women [8, 16, 19, 20].

While most of the studies were homogenous, geo-political differences and constantly shifting cultural ideologies make findings difficult to generalize. Other limitations include not drawing a long-term impact on infants and young children’s oral health. Our review also focused on only peer-reviewed original studies published in English; as such, we may miss valuable opinion editorials or interviews conducted in French or other local languages.

## Future directions

The studies in this review established that dental health awareness is suboptimal among African pregnant women, and certain beliefs act as barriers to them utilizing dental care or seeking oral health care during pregnancy. Oral health education is an effective tool that can bridge the knowledge gap among pregnant women. Therefore, oral health education should be employed to increase knowledge and address beliefs that prevent dental care-seeking behavior in pregnancy. Multidisciplinary oral health education may be required so that all care providers involved in caring for pregnant women during pregnancy point them to seeking oral health care, making it a crucial component of pregnancy care, as it could benefit the outcome of their pregnancy.

Incorporating local stories and oral health folklore [33] around traditional pregnancy beliefs can improve pregnant women’s oral health attitudes. Delivering oral health talks at the grassroots level, particularly in primary health centers and antenatal clinics, provides valuable opportunities to educate mothers. These settings serve as key points where mothers learn and refine their knowledge on caring for their unborn children, both now and in the future. African governments, stakeholders, and policymakers need to ensure standard dental care facilities are easily accessible. Given that this review focused solely on Nigeria, Sudan, and South Africa, the findings may not fully represent the broader context of Sub-Saharan Africa, thereby limiting their generalizability. Future studies should also look into novel, cost-effective strategies to help low-income countries achieve the WHO target of universal oral health by 2030.

## Conclusion

This systematic review provides a holistic evaluation of the utilization of oral health services among pregnant women in Africa. There is a need for a behavioral shift that stems from grassroots education and the provision of accessible oral health facilities. Despite some limitations such as limited generalizability, the reviewed studies demonstrated a low risk of bias and consistent findings. Pregnancy is a critical period, and poor oral health can adversely affect birth outcomes. We found insufficient knowledge of misconceptions that deter women from seeking dental care during pregnancy. To address these issues, targeted oral health education must improve awareness and overcome barriers to seeking care. We also call for future studies to address cost-effective interventions that can improve oral health-seeking behaviors of pregnant women in Africa.

## Electronic supplementary material

Below is the link to the electronic supplementary material.

Supplementary Material 1

## Data Availability

The datasets used and/or analyzed during the current study are available from the corresponding author on reasonable request.

## Abbreviations

SSA: Sub-Saharan Africa
PRISMA: Preferred Reporting Items for Systematic Reviews and Meta-Analyses
PEO: Population, Exposure, and Outcome
AJOL: African Journals Online
DOAJ: Directory of Open Access Journals
CSV: Comma Separated Values
LASUTH: Lagos State University Teaching Hospital
