# Supplementary material for: Oral health knowledge, perceptions and attitudes of pregnant women in Sub-Saharan Africa: a systematic review
Source: BMC Oral Health. 2025 Jun 6;25:937. doi: 10.1186/s12903-025-06249-y (PMC12144732; doi:10.1186/s12903-025-06249-y)
Supplement: Supplementary file 1 — Supplementary Material 1 [file 12903_2025_6249_MOESM1_ESM.docx]

**Oral Health Knowledge, Perceptions and Attitudes of Pregnant Women in Sub-Saharan Africa: A Systematic Review- Search Template Guide.**

Study design

The study will be a Systematic review.

Inclusion Criteria

· Original general and oral health (medical) articles focusing on the knowledge, perception and attributes of pregnant women to oral health changes**.**

· Published in the English Language.

Exclusion Criteria

· Letters, Opinions, Reviews and Comments

P- Pregnant women in sub-Saharan Africa

E- Exposure is the knowledge, perceptions, and attitudes toward oral health

O- Outcome is understanding, beliefs, and behaviors related to oral health care during pregnancy

Data collection procedure- The study will be done by retrieving articles from PubMed, Google Scholar, Science Direct, DOAJ (Directory of Open Access Journals), AJOL (African Journals Online), Cochrane Library and using relevant BOOLEAN strings from our inclusion and exclusion criteria.

Template for Developing Search Strategy

|  | Concept 1 | Concept 2 |
| --- | --- | --- |
| Key Concept | **Pregnancy** | **Oral helth** |
| Free Text Term/Natural Language Term | - Pregnancy - Mothers - Pregnant women - Maternal-Fetal Relations | - Dental Clinics - Dental Health Surveys - [Diagnosis, Oral](https://www.ncbi.nlm.nih.gov/mesh/68003945) - Mouth Diseases - Mouth Rehabilitation |
| MeSH Term | - Pregnant women | - Oral Health |
| Type of Study (other filters) | English Language  Original Articles  Females  Clinical Study | English Language  Original Articles  Females  Clinical Study |

PUBMED: (("oral health"[MeSH Terms] OR ("oral"[All Fields] AND "health"[All Fields]) OR "oral health"[All Fields]) AND ("case reports"[Publication Type] OR "clinical study"[Publication Type]) AND (("pregnant"[All Fields] OR "pregnants"[All Fields]) AND ("case reports"[Publication Type] OR "clinical study"[Publication Type])) AND (("africa south of the sahara"[MeSH Terms] OR ("africa"[All Fields] AND "south"[All Fields] AND "sahara"[All Fields]) OR "africa south of the sahara"[All Fields] OR ("sub"[All Fields] AND "saharan"[All Fields] AND "africa"[All Fields]) OR "sub saharan africa"[All Fields]) AND ("case reports"[Publication Type] OR "clinical study"[Publication Type]))) AND (casereports[Filter] OR clinicalstudy[Filter]) as at **30th March, 2025**

Results= **85**

GOOGLE SCHOLAR: ("Oral*" OR "dent*" OR "Oro*" OR "tooth*" OR "teeth*") AND ("preg*" OR "wom*") AND ("africa*")

Results= **19**

SCIENCE DIRECT[:](https://www.sciencedirect.com/search?qs=Oral%20health%20changes%20during%20pregnancy%20in%20african%20women&articleTypes=FLA&lastSelectedFacet=articleTypes)

Results= **28**

[DOAJ:](https://doaj.org/search/articles?ref=homepage-box&source=%7B%22query%22%3A%7B%22query_string%22%3A%7B%22query%22%3A%22Oral%20health%20changes%20during%20pregnancy%20in%20africa%22%2C%22default_operator%22%3A%22AND%22%7D%7D%2C%22size%22%3A%22100%22%2C%22track_total_hits%22%3Atrue%7D)

Results= **3**

[AJOL:](https://www.ajol.info/index.php/ajol/Gsearch/google?q=Oral+health+changes+during+pregnancy+in+african+women#gsc.tab=0&gsc.q=Oral%20health%20changes%20during%20pregnancy%20in%20african%20women&gsc.page=1)

Results: **24**

Cochrane Library: MeSH descriptor: [Oral Health] MeSH descriptor: [Pregnancy]

= **26**

Finally:

Two independent reviewers manually cross-checked each article for relevance.
